# Association Between Early Spontaneous Post‐Thrombectomy Blood Pressure Reduction and Clinical Outcomes in Large Vessel Occlusion Stroke

**DOI:** 10.1002/brb3.70677

**Published:** 2025-07-07

**Authors:** Jianwen Bu, Guosen Bu, Yang Zhang

**Affiliations:** ^1^ Department of Trauma Orthopedic Surgery First Affiliated Hospital of Xinjiang Medical University Urumqi Xinjiang China; ^2^ Department of Neurology First Affiliated Hospital of Xinjiang Medical University Urumqi Xinjiang China; ^3^ Department of Emergency Surgery, Emergency Trauma Center First Affiliated Hospital of Xinjiang Medical University Urumqi Xinjiang China

**Keywords:** blood pressure, large vessel occlusion, outcome, stroke, thrombectomy

## Abstract

**Objective:**

To investigate the association between early spontaneous blood pressure (BP) reductions after thrombectomy, without pharmacological intervention, and clinical outcomes in patients with acute anterior circulation large vessel occlusion (LVO) stroke.

**Methods:**

Consecutive patients with anterior circulation LVO stroke who underwent thrombectomy at a single center between January 2021 and June 2024 were retrospectively analyzed. Blood pressure (BP) measurements were obtained upon arrival at the angiography suite and within half an hour post‐thrombectomy, along with the reductions in BP before and after the thrombectomy (ΔBP). Multivariate logistic regression was used to identify independent predictors of favorable outcome (90‐day modified Rankin Scale score of 0–2), while receiver operating characteristic (ROC) curves evaluated predictive performance.

**Results:**

Among 484 patients, 41.5% achieved a favorable outcome. Greater systolic ΔBP (ΔSBP) reduction (per 10 mmHg increase) (adjusted odds ratio [OR], 1.12; 95% confidence interval [CI], 1.06–1.18) and nadir SBP within half an hour post‐thrombectomy (per 10 mmHg increase) (adjusted OR, 0.98; 95% CI, 0.97–0.99) were independently associated with favorable outcome. In addition, National Institutes of Health Stroke Scale scores, collateral scores, successful recanalization, hemorrhagic transformation, and symptomatic intracranial hemorrhage were also associated with the outcome (P < 0.05). ΔSBP and nadir SBP within half an hour post‐thrombectomy predicted favorable outcomes with individual AUCs of 0.81 and 0.76, respectively, and a combined AUC of 0.88 (sensitivity 82.4%, specificity 85.7%, PPV 78.9%).

**Conclusion:**

Early spontaneous post‐thrombectomy SBP reduction independently predicts favorable outcome in LVO, with improved predictive accuracy when combined with nadir SBP early post‐thrombectomy.

## Graphical abstract

Early spontaneous systolic blood pressure (SBP) reduction after thrombectomy—without pharmacologic intervention—was independently associated with favorable outcomes in patients with anterior circulation large vessel occlusion stroke. Combining ΔSBP with nadir SBP within 30 min post‐procedure significantly improved predictive accuracy for 90‐day functional recovery.

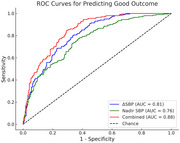

## 1 Introduction

Endovascular thrombectomy (EVT) has become the standard of care for acute large vessel occlusion (LVO) stroke, with technical success rates approaching 90% (Turc et al. 2019; Lapergue et al. 2021). Despite these advancements, more than half of patients undergoing EVT still experience significant functional disability (Majoie et al. 2023; Weyland et al. 2022). Among the myriad factors influencing functional outcomes, perioperative blood pressure (BP) management has drawn increasing attention due to its potential impact on recovery and complications.

Retrospective studies demonstrated a strong association between perioperative BP levels and functional outcomes following EVT. Specifically, elevated BP levels after thrombectomy have been linked to an increased risk of hemorrhagic transformation (HT) and poor outcomes (Ding et al. 2020; Gigliotti et al. 2021; Liu et al. 2024; Liu et al. 2021). The BP‐TARGET trial was the first randomized controlled trial (RCT) to investigate the effects of post‐thrombectomy BP control on clinical outcomes (Mazighi et al. 2021). It revealed that intensive systolic BP control (100–129 mmHg) did not significantly reduce the incidence of HT compared to standard systolic BP control (130–185 mmHg). Subsequent clinical trials, including ENCHANTED2/MT, BEST II, and OPTIMAL BP, have corroborated these findings, showing no significant benefit of aggressive BP lowering over conventional BP management (Yang et al. 2022; Mistry et al. 2023; Nam et al. 2021). Moreover, excessive BP reduction may compromise cerebral perfusion, particularly in ischemically vulnerable brain regions, potentially leading to infarct core expansion (Yang et al. 2022; Mistry et al. 2023; Nam et al. 2021; Jiang et al. 2024).

However, these studies have predominantly focused on BP management through active interventions. The relationship between early spontaneous BP reductions, in the absence of external interventions, and clinical outcomes in patients undergoing EVT has been minimally explored. This study aimed to investigate the association between early spontaneous BP reductions after EVT, in the absence of pharmacological intervention, and clinical outcomes in patients with acute LVO stroke.

## 2 Methods

### 2.1 Study Population

Since 2018, our center has employed an electronic follow‐up system to prospectively record data on all patients undergoing EVT for acute LVO stroke. This system captures comprehensive information, including epidemiological details, comorbidities, medication history, diagnostic and treatment timelines, thrombectomy specifics, hospitalization records, and 90‐day functional outcomes. Trained stroke nurses conducted structured telephone interviews to obtain 90‐day outcomes, while other data were extracted at discharge from medical records, imaging studies, and laboratory systems and integrated into the database. We retrospectively analyzed data from this database to identify consecutive patients who underwent EVT for terminal internal carotid artery (ICA) or first‐segment middle cerebral artery (MCA) occlusions between January 2021 and June 2024 (n = 562). Treatment decisions within 6 h of symptom onset were based on the Alberta Stroke Program Early CT Score (ASPECTS), while those within 6–24 h relied on CT perfusion findings (Liu et al. 2023). Inclusion criteria were (1) age ≥ 18 years and (2) symptom onset within 24 h. Exclusion criteria included (i) a pre‐stroke modified Rankin Scale (mRS) score ≥ 2 (n = 5) and (ii) administration of sedatives, general anesthesia, or antihypertensive agents during thrombectomy (n = 36) in order to avoid pharmacological interference with BP measurements. All included patients underwent thrombectomy under local anesthesia only, without the use of sedation or general anesthesia, (iii) missing BP data at angiography suite arrival or post‐thrombectomy (n = 21), (iv) absence of follow‐up imaging (n = 11), and (v) missing 90‐day outcomes (n = 5).

### 2.2 Thrombectomy Procedures

All thrombectomies were performed in accordance with established clinical guidelines and protocols. Patients within the thrombolytic time window and without contraindications received a full‐dose of intravenous recombinant tissue plasminogen activator (rt‐PA) prior to the thrombectomy. During thrombectomy, an 8F guiding catheter or a 7F long sheath was advanced under fluoroscopic guidance to the proximal segment of the occluded target vessel. The choice of thrombectomy technique was determined by the neurointerventionalist and included the following approaches: Stent retriever thrombectomy (Solitaire FR, Medtronic, Dublin, Ireland; Trevo, Stryker, Kalamazoo, MI, USA). Aspiration thrombectomy (Sofia, MicroVention, Aliso Viejo, CA, USA; ACE60/68, Penumbra, Alameda, CA, USA). Combined techniques, the simultaneous use of stent retrievers and aspiration catheters to enhance recanalization efficacy. In cases where stroke etiology was attributed to large‐artery atherosclerosis with associated stenosis, rescue therapy measures—such as balloon angioplasty, stent angioplasty, and intraoperative administration of tirofiban (a glycoprotein IIb/IIIa receptor inhibitor)—may be employed. Twenty‐four hours post‐thrombectomy, patients without evidence of HT or large infarct were transitioned to dual antiplatelet therapy with aspirin (100 mg daily) and clopidogrel (75 mg daily). In contrast, if intracranial HT or substantial infarct burden was present, antiplatelet therapy was either deferred or limited to single‐agent therapy based on clinical assessment and follow‐up imaging.

### 2.3 BP Management and Collection

BP management followed the guidelines established by the American Heart Association (AHA) and the American Stroke Association (ASA) (Powers et al. 2019). Pre‐thrombectomy BP was maintained below 185/110 mmHg, while intra‐thrombectomy and post‐thrombectomy BP was controlled below 180/105 mmHg. Patients requiring intravenous antihypertensives during the procedure or within the first 30 min post‐thrombectomy were excluded to ensure the analysis reflected spontaneous BP changes. Upon arrival at the angiography suite, BP was measured using an upper‐arm BP monitoring device. Subsequently, BP measurements were taken every 5 min until the procedure was completed and the patient exited the angiography suite. The recorded BP parameters included systolic BP (SBP), diastolic BP (DBP), and mean arterial pressure (MAP) measured at angiography suite arrival and within half an hour post‐thrombectomy. The lowest BP recorded within half an hour post‐thrombectomy was designated as the post‐procedural BP for statistical analysis. BP change (ΔBP) was defined as the difference between BP at angiography suite arrival and the lowest BP within half an hour post‐thrombectomy.

### 2.4 Outcomes Evaluation

The post‐thrombectomy imaging follow‐up protocol included a non‐contrast CT (NCCT) scan performed within 24 h after thrombectomy or immediately upon neurological deterioration. If intracranial hyperdensity was observed on the initial follow‐up imaging, an additional CT scan was performed 24–48 h later to distinguish contrast extravasation from HT (Payabvash et al. 2014). A follow‐up scan was conducted on post‐procedural days 5–7 to confirm the final infarct volume. Recanalization success was assessed using the modified Thrombolysis in Cerebral Infarction (mTICI) scale, with mTICI ≥ 2b considered successful recanalization. A favorable outcome was defined as 90‐day mRS scores ≤ 2. HT was classified according to the Heidelberg Bleeding Classification (von Kummer et al. 2015). Symptomatic intracranial hemorrhage (sICH) was defined as parenchymal hematoma, subarachnoid hemorrhage, or intraventricular hemorrhage associated with neurological deterioration. Neurological deterioration was defined by any of the following criteria: (i) an increase of ≥ 4 points in the National Institutes of Health Stroke Scale (NIHSS) score compared to pre‐deterioration levels, (ii) an increase of ≥ 2 points in any single NIHSS category, (iii) clinical worsening requiring significant medical or surgical intervention, or (iv) unexplained worsening. Functional outcomes were assessed using the mRS at 90‐day, with mRS scores of 0–2 defined as favorable outcomes. In addition to standard outcome measures, early neurological deterioration (END) was analyzed as a distinct early adverse event. END was defined post hoc as a ≥4‐point increase in NIHSS score within 24 h after thrombectomy, irrespective of imaging findings (Nam et al. 2021).

### 2.5 Statistical Analysis

Continuous data are presented as median and interquartile range (IQR) or mean and standard deviation (SD), as appropriate. Statistical comparisons were performed using Student's t‐test for normally distributed variables and the Mann‐Whitney U test for non‐normally distributed variables. Categorical data were expressed as frequencies and percentages, and comparisons were conducted using the chi‐square test. Logistic regression analysis was employed to assess the association between variables and functional outcomes. Variables demonstrating an association with functional outcomes in univariate analysis (P < 0.05) were included in the multivariate logistic regression model. Results are reported as adjusted odds ratios (aOR) with 95% confidence intervals (CI). Receiver operating characteristic (ROC) curves were used to determine the cutoff values and accuracy of predictors for functional outcomes.

Additional analyses were performed to explore the relationship between reperfusion degree and spontaneous postprocedural BP reductions. Patients were categorized according to detailed mTICI scores (0–1, 2a, 2b, 2c, and 3). ΔSBP values and nadir SBP within half an hour post‐thrombectomy were compared across these five groups using the Kruskal‐Wallis test. A sensitivity analysis was also conducted by incorporating reperfusion grade, baseline core infarct volume (rCBF < 30%), baseline NIHSS, collateral score, the presence of HT, and sICH into a multivariable logistic regression model to verify the independent predictive value of spontaneous BP reduction for favorable clinical outcomes. A two‐sided P‐value < 0.05 was considered statistically significant. Statistical analyses were conducted using SPSS version 27 (IBM Corp., Armonk, NY, USA) and R version 3.3.3 (R Foundation for Statistical Computing, Vienna, Austria), following standard practices for data analysis.

## 3 Results

**FIGURE 1:**
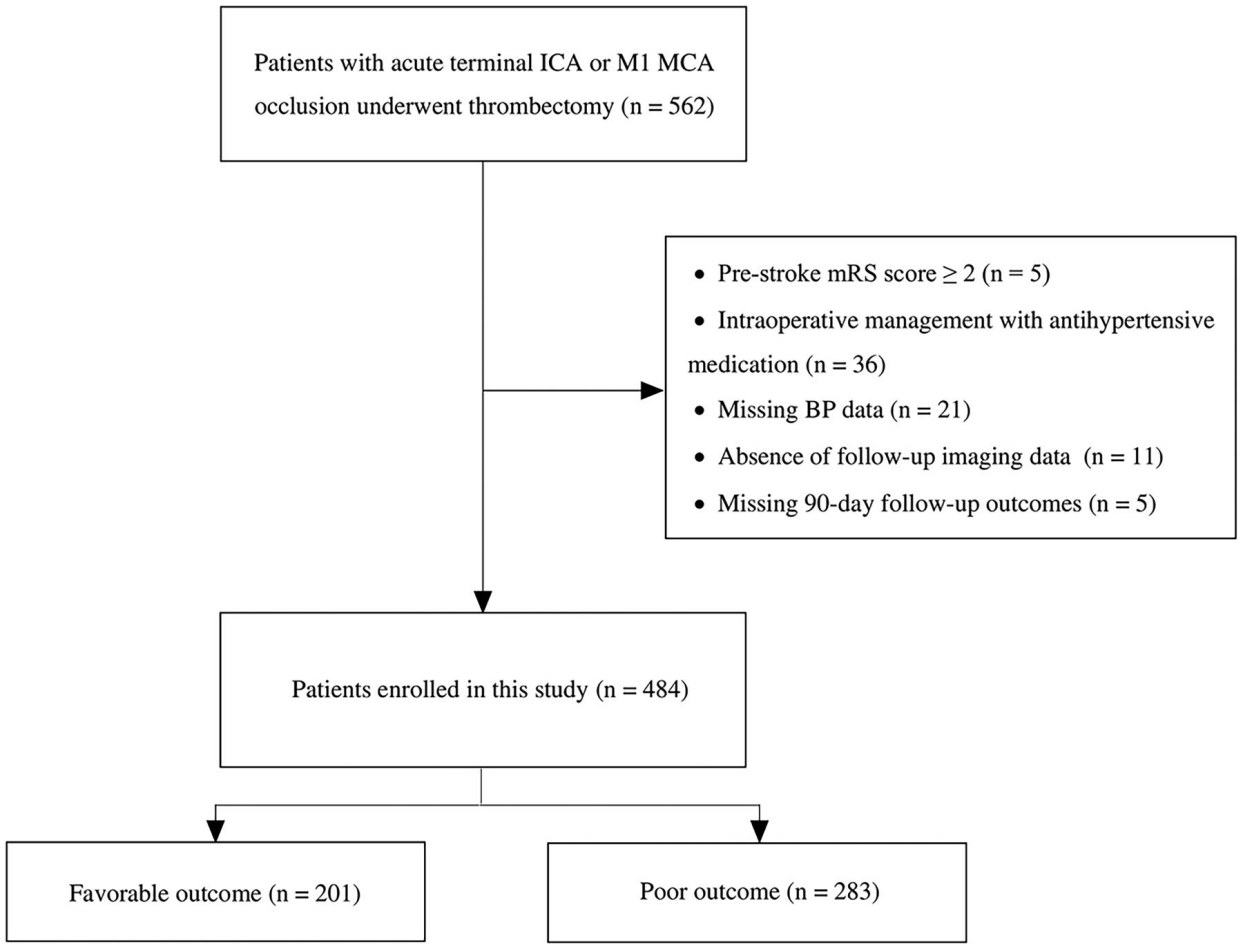
Flow chart of patients. ICA, internal carotid artery; MCA, middle cerebral artery; mRS, modified Rankin Scale; BP, blood pressure.

A total of 484 patients were included during the study period (Figure 1). The mean age was 72.2 ± 11.9 years, with 54.2% being male. The median baseline NIHSS score was 13 (IQR, 9–17), the median ASPECTS was 8 (IQR, 8–9), and the median time from symptom onset to groin puncture was 231 min (IQR, 172–368). Successful reperfusion was achieved in 85.7% of cases, while the incidences of HT and sICH were 24.8% and 7.6%, respectively. Patients with favorable outcomes had lower NIHSS scores (median 11 vs. 14, P < 0.001), higher collateral scores (69.2% vs. 40.3%, P < 0.001), greater ΔSBP reductions (median 26 mmHg [IQR, 22–31] vs. 11 mmHg [IQR, 6–19], P < 0.001), and lower nadir SBP within 30 min post‐procedure (median 132 mmHg [IQR, 125–144] vs. 147 mmHg [IQR, 131–159], P = 0.001) (Table 1). Successful reperfusion was more frequent (92.5% vs. 80.9%, P < 0.001), while HT (16.4% vs. 30.7%, P < 0.001) and sICH (1.5% vs. 12.0%, P < 0.001) were less common in this group.

Multivariate logistic regression identified several characteristics significantly associated with a favorable outcome: ΔSBP reduction (per 10 mmHg increase) (adjusted OR, 1.12; 95% CI, 1.06–1.18; P = 0.002), nadir SBP post‐thrombectomy (per 10 mmHg increase) (adjusted OR, 0.98; 95% CI, 0.97–0.99; P = 0.021), baseline NIHSS scores (adjusted OR, 0.88; 95% CI, 0.82–0.95; P < 0.001), collateral scores (adjusted OR, 2.35; 95% CI, 1.60–3.45; P < 0.001), and successful reperfusion (adjusted OR, 3.12; 95% CI, 1.70–5.72; P < 0.001) (Table 2). Conversely, HT (adjusted OR, 0.48; 95% CI, 0.29–0.80; P = 0.005) and sICH (adjusted OR, 0.18; 95% CI, 0.04‐0.60; P = 0.004) were inversely associated with favorable outcome.

**FIGURE 2:**
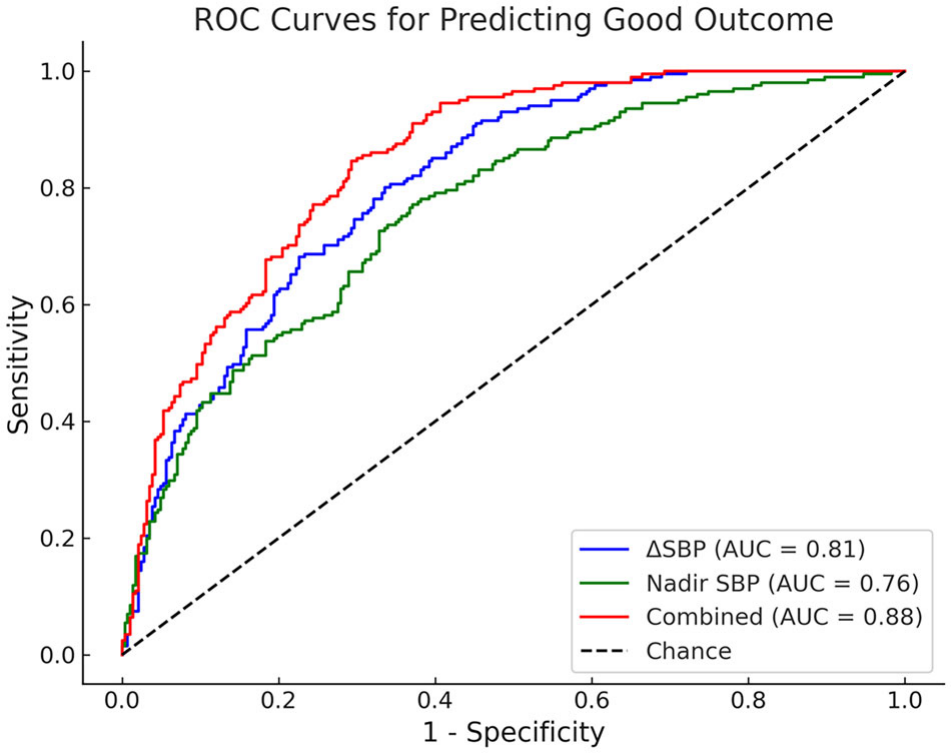
ROC curve was performed to assess the accuracy of the model in predicting favorable outcomes. The ΔSBP demonstrated an AUC of 0.81, the nadir SBP within the first half‐hour post‐thrombectomy achieved an AUC of 0.76, and their combined use yielded an AUC of 0.88. ROC, receiver operating characteristic; ΔSBP, change in systolic blood pressure; AUC, area under the curve; SBP, systolic blood pressure.

ROC analysis showed ΔSBP had an AUC of 0.81 (95% CI, 0.78–0.85) with an optimal cutoff of 23 mmHg, yielding 76.5% sensitivity, 78.9% specificity, 70.3% positive predictive value (PPV), and 82.7% negative predictive value (NPV) (Figure 2). Nadir SBP post‐thrombectomy achieved an AUC of 0.76 (95% CI, 0.72–0.80) with an optimal cutoff of 140 mmHg, yielding 71.6% sensitivity, 74.3% specificity, 68.2% PPV, and 77.5% NPV. Combined, two improved the AUC to 0.88 (95% CI, 0.84–0.91), with 82.4% sensitivity, 85.7% specificity, 78.9% PPV, and 87.6% NPV. Compared to ΔSBP alone, the combined model reduced the false‐positive rate from 21.1% to 14.3% and the false‐negative rate from 23.5% to 17.6%.

**FIGURE 3:**
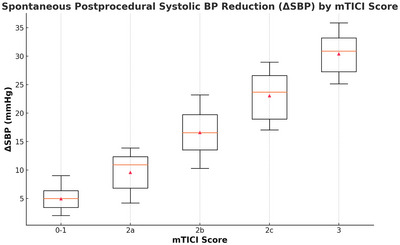
Association between spontaneous postprocedural ΔSBP and detailed mTICI scores. Boxplots illustrating ΔSBP across five reperfusion grades (mTICI 0–1, 2a, 2b, 2c, and 3). A significant trend was observed, with greater spontaneous ΔSBP reductions in patients achieving higher mTICI scores (P = 0.002). ΔSBP, change in systolic blood pressure; mTICI, modified Thrombolysis in Cerebral Infarction.

**FIGURE 4:**
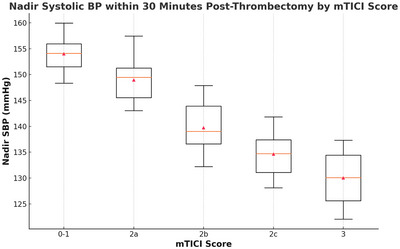
Association between nadir SBP within half an hour post‐thrombectomy and mTICI scores. Boxplots illustrating nadir SBP across five reperfusion grades (mTICI 0–1, 2a, 2b, 2c, and 3). A significant trend was observed, with lower nadir SBP values associated with higher mTICI scores (P = 0.003). SBP, systolic blood pressure; mTICI, modified Thrombolysis in Cerebral Infarction.

There was a significant association between the degree of reperfusion and both ΔSBP and nadir SBP following thrombectomy. Specifically, ΔSBP increased progressively with higher mTICI scores (P = 0.002, Kruskal–Wallis test), with median values of 5 mmHg (IQR, 2–10) for mTICI 0–1, 8 mmHg (IQR, 4–14) for 2a, 16 mmHg (IQR, 10–24) for 2b, 22 mmHg (IQR, 17–29) for 2c, and 30 mmHg (IQR, 25–36) for mTICI 3 (Figure 1, 2, 3). Conversely, nadir SBP within 30 min post‐thrombectomy demonstrated an inverse association with reperfusion grade (P = 0.003, Kruskal–Wallis test), with median values of 156 mmHg (IQR, 148–162) for mTICI 0–1, 152 mmHg (IQR, 142–158) for 2a, 141 mmHg (IQR, 132–148) for 2b, 134 mmHg (IQR, 128–142) for 2c, and 129 mmHg (IQR, 122–138) for mTICI 3 (Figure 4). In a multivariable logistic regression model adjusting for potential variables, both ΔSBP and nadir SBP remained independently associated with favorable 90‐day functional outcomes. Specifically, ΔSBP (per 10 mmHg increase) was associated with an adjusted OR of 1.10 (95% CI, 1.04–1.16; P = 0.001), and nadir SBP (per 10 mmHg increase) with an adjusted OR of 0.94 (95% CI, 0.91–0.98; P = 0.002).

Comparison between patients with and without HT revealed that those who developed HT had significantly higher baseline NIHSS scores, lower ASPECTS, worse collateral status, larger infarct core volumes, smaller ΔSBP, and higher nadir SBP within 30 min post‐thrombectomy (all P < 0.05, Supplemental Table 1). In the multivariable logistic regression analysis (Supplemental Table 2), greater ΔSBP was independently associated with a lower risk of HT (adjusted OR = 0.72 per 10 mmHg increase; 95% CI, 0.59–0.89; P = 0.002). Conversely, higher nadir SBP was associated with an increased risk of HT (adjusted OR = 1.32 per 10 mmHg increase; 95% CI, 1.12–1.62; P = 0.001). Other significant predictors of HT included baseline NIHSS score, collateral score, ASPECTS, and infarct core volume. Besides, a total of 41 patients (8.5%) experienced END within 24 h post‐thrombectomy. In multivariate logistic regression adjusting for baseline NIHSS score, collateral status, reperfusion grade, and hemorrhagic complications, greater ΔSBP was independently associated with a lower risk of END (adjusted OR per 10 mmHg increase: 0.88; 95% CI: 0.80–0.96; P = 0.005), while higher nadir SBP was associated with an increased risk of END (adjusted OR per 10 mmHg increase: 1.11; 95% CI: 1.06–1.18; P = 0.002).

## 4 Discussion

This study aimed to investigate the association between early spontaneous BP reductions after EVT, in the absence of pharmacological intervention, and clinical outcomes in patients with acute LVO stroke. Our results demonstrate that greater spontaneous reductions in SBP within the first 30 min post‐thrombectomy are significantly associated with improved functional outcomes. Notably, the nadir SBP observed during this immediate post‐procedural period independently predicted favorable outcomes, and its combination with early ΔSBP substantially improved predictive accuracy (AUC = 0.88). These findings underscore the prognostic importance of early spontaneous BP dynamics as potential noninvasive indicators of successful reperfusion and clinical recovery.

**TABLE 1:** Comparison of patient characteristics between favorable and poor outcome groups.

Characteristics	Favorable outcome (n = 201)	Poor outcome (n = 283)	P values
Age, mean (SD), y	71.7 ± 12.5	72.5 ± 11.6	0.674
Male, n (%)	109 (54.2)	146 (51.6)	0.567
Hypertension, n (%)			0.884
No prior hypertension	73 (36.3)	105 (37.1)	
Controlled hypertension	111 (55.2)	151 (53.4)	
Uncontrolled hypertension	17 (8.5)	27 (9.5)	
Diabetic mellitus, n (%)	32 (15.9)	45 (15.9)	0.995
Coronary heart disease, n (%)	24 (11.9)	40 (14.1)	0.483
Atrial fibrillation, n (%)	20 (10.0)	39 (13.9)	0.204
Previous stroke, n (%)	29 (14.4)	33 (11.7)	0.447
New York Heart Association Class III–IV, n (%)	6 (3.0)	14 (4.9)	0.403
Chronic kidney disease, n (%)	15 (7.6)	27 (9.5)	0.525
Autoimmune diseases, n (%)	5 (2.5)	9 (3.2)	0.863
BP‐altering medications, n (%)	21 (10.4)	34 (12.0)	0.697
Baseline NIHSS, median (IQR)	11 (8‐15)	14 (10‐18)	< 0.001
Time from onset to groin puncture, median (IQR), min	206 (161‐343)	243 (186‐395)	0.274
SBP at angiosuite arrival, median (IQR), mmHg	165 (146‐173)	158 (144‐169)	0.852
DBP at angiosuite arrival, median (IQR), mmHg	95 (84‐106)	89 (79‐103)	0.414
MAP, angiosuite arrival, median (IQR), mmHg	117 (104‐127)	112 (100‐125)	0.658
Nadir SBP within half an hour post‐thrombectomy, median (IQR), mmHg	132 (125‐144)	147 (131‐159)	0.001
Nadir DBP within half an hour post‐thrombectomy, median (IQR), mmHg	84 (74‐90)	89 (76‐97)	0.594
Nadir MAP, within half an hour post‐thrombectomy, median (IQR), mmHg	100 (90‐108)	107 (93‐116)	0.560
ΔSBP, median (IQR), mmHg	26 (22‐31)	11 (6‐19)	< 0.001
ΔDBP, median (IQR), mmHg	10 (5‐16)	6 (2‐10)	0.141
ΔMAP, median (IQR), mmHg	14 (7‐21)	7 (5‐16)	0.156
Collateral score, n (%)			< 0.001
0–1	62 (30.8)	169 (59.7)	
2–3	139 (69.2)	114 (40.3)	
Occlusion site, n (%)			0.439
Terminal ICA	83 (41.3)	107 (37.8)	
M1 MCA	118 (58.7)	176 (62.2)	
ASPECTS, median (IQR), min	9 (9‐10)	8 (7‐9)	
Stroke etiology, n (%)			0.483
Large artery atherosclerosis	80 (39.8)	91 (32.2)	
Cardioembolism	68 (33.8)	106 (37.5)	
Other determined	4 (2.0)	5 (1.8)	
Undetermined	49 (24.4)	81 (28.6)	
rCBF < 30%, median (IQR), mL	15 (9‐29)	26 (18‐35)	0.413
Tmax > 6s volume, median (IQR), mL	84 (68‐104)	92 (71‐108)	0.865
mTICI ≥ 2b, n (%)	186 (92.5)	229 (80.9)	< 0.001
mTICI ≥ 2c, n (%)	146 (72.6)	147 (51.9)	< 0.001
mTICI = 3, n (%)	120 (59.7)	112 (40.0)	< 0.001
HT, n (%)	33 (16.4)	87 (30.7)	< 0.001
sICH, n (%)	3 (1.5)	34 (12.0)	< 0.001

*Notes*: P‐values were calculated using Pearson's chi‐square test or Fisher's exact test as appropriate. Values equal to or near 1.000 indicate results from Fisher's exact test with no observed group differences.

Abbreviations: ASPECTS, Alberta Stroke Program Early CT Score; DBP, diastolic blood pressure; HT, hemorrhagic transformation; ICA, internal carotid artery; IQR, interquartile range; MAP, mean arterial pressure; MCA, middle cerebral artery; mTICI, Modified Thrombolysis in Cerebral Infarction; NIHSS, National Institutes of Health Stroke Scale; rCBF, relative cerebral blood flow; SBP, systolic blood pressure; SD, standard deviation; sICH, symptomatic intracranial hemorrhage; Tmax, time‐to‐maximum; ΔDBP, change in diastolic blood pressure; ΔMAP, change in mean arterial pressure; ΔSBP, change in systolic blood pressure.

**TABLE 2:** Univariate and multivariate regression analysis of characteristics associated with favorable outcome.

	n	Unadjusted		Adjusted	
		OR (95% CI)	P	OR (95% CI)	P
Baseline NIHSS (per point increase)	484	0.85 (0.80‐0.90)	< 0.001	0.88 (0.82‐0.95)	< 0.001
nadirSBP within half an hour post‐procedure (per 10 mmHg increase)	484	0.97 (0.96‐0.99)	0.001	0.98 (0.97‐0.99)	0.021
ΔSBP (per 10 mmHg decrease)	484	1.15 (1.10‐1.21)	< 0.001	1.12 (1.06‐1.18)	0.002
Collateral score					
0‐1	231	Ref		Ref	
2‐3	253	2.86 (2.00‐4.09)	< 0.001	2.35 (1.60‐3.45)	< 0.001
Successful reperfusion	415	3.50 (2.00‐6.12)	< 0.001	3.12 (1.70‐5.72)	< 0.001
HT	120	0.43 (0.27‐0.68)	< 0.001	0.48 (0.29‐0.80)	0.005
sICH	37	0.12 (0.03‐0.40)	< 0.001	0.18 (0.04‐0.60)	0.004

Abbreviations: CI, confidence interval; HT, hemorrhagic transformation; NIHSS, National Institutes of Health Stroke Scale; OR, odds ratio; SBP, systolic blood pressure; sICH, symptomatic intracranial hemorrhage.; ΔSBP, change in systolic blood pressure.

Previous studies have predominantly focused on pharmacologically managed BP after EVT, with mixed results regarding optimal BP targets. Elevated BP and high BP variability post‐EVT have been linked to increased risks of hemorrhagic transformation and poor functional outcomes, prompting recommendations for tighter BP control (Ding et al. 2020; Gigliotti et al. 2021; Liu et al. 2024; Liu et al. 2021; Palaiodimou et al. 2024). However, several randomized trials, such as ENCHANTED2/MT and OPTIMAL‐BP, found limited or no benefit with intensive BP lowering, partly because achieved BP differences between treatment groups were modest (Mazighi et al. 2021; Yang et al. 2022; Mistry et al. 2023; Nam et al. 2021). Notably, spontaneous BP declines post‐procedure in many patients might have masked the effects of intensive pharmacological BP management. In contrast, our analysis excluded pharmacologic confounders, specifically examining early spontaneous BP reductions. Anadani et al. demonstrated a beneficial association between spontaneous SBP reductions and favorable outcomes, but their analysis extended over 24 h and included patients receiving antihypertensive treatments, potentially introducing confounders (Anadani et al. 2020). Dias et al. identified spontaneous SBP reduction as an independent predictor of early neurological recovery but did not evaluate nadir SBP separately (Carvalho Dias et al. 2020). Building upon these studies, we uniquely identify the immediate post‐thrombectomy nadir SBP as an additional independent prognostic marker, emphasizing the potential utility of early, spontaneous BP patterns in clinical decision‐making.

Several mechanisms may explain the association between spontaneous BP reduction and improved outcomes. First, acute ischemic stroke and LVO often lead to elevated BP as a compensatory response to maintain cerebral perfusion in the context of impaired autoregulation (Reddy et al. 2023). Successful reperfusion alleviates ischemic stress, allowing BP to spontaneously normalize (Rusanen et al. 2015). This natural normalization avoids the risks associated with externally induced BP lowering, such as hypoperfusion and infarct core expansion. In contrast, persistent hypertension after EVT may indicate insufficient cerebral perfusion or microvascular dysfunction, even when macrovascular reperfusion is achieved (Jiang et al. 2017). Second, findings from several RCTs have demonstrated that aggressive BP lowering does not improve outcomes and may even be associated with adverse effects (Mazighi et al. 2021; Yang et al. 2022; Mistry et al. 2023; Nam et al. 2021). Intensive BP reduction can compromise collateral flow to penumbral regions, particularly in cases of incomplete reperfusion or poor collateral circulation (Mazighi et al. 2021; Yang et al. 2022; Mistry et al. 2023; Nam et al. 2021; Rusanen et al. 2015). Conversely, spontaneous and moderate BP reduction may maintain adequate perfusion in these vulnerable regions, preventing hypoperfusion‐related damage while minimizing the risk of HT. Our additional analyses demonstrated that patients with higher mTICI grades exhibited greater spontaneous reductions in SBP and lower nadir SBP values within 30 min following thrombectomy. These changes likely reflect the restoration of cerebral perfusion and physiological homeostasis after successful reperfusion. This adaptive response—marked by pronounced BP declines and lower minimum SBP—may result from improved vessel patency, reduced ischemic stress, and a more favorable microvascular environment. Collectively, these findings highlight the prognostic value of early, spontaneous BP trajectories as noninvasive markers of reperfusion quality and clinical outcome following endovascular treatment.

ΔSBP and nadir SBP post‐thrombectomy are easily measurable parameters that can be integrated into routine post‐thrombectomy monitoring. Unlike intensive BP management strategies that require frequent adjustments, monitoring spontaneous BP trends is a non‐invasive method that reflects the patient's physiological status. Importantly, ΔSBP and nadir SBP thresholds can inform risk stratification and clinical decision‐making. For example, patients with a ΔSBP decrease of more than 23 mmHg or a nadir SBP less than 140 mmHg within 30 min after EVT are more likely to have a good outcome. Incorporating these thresholds into post‐thrombectomy care algorithms may improve the accuracy of resource allocation and improve patient outcomes.

This study has several limitations. First, as a single‐center retrospective analysis, selection bias is unavoidable, and the findings may not be generalizable to broader populations. Second, all included patients underwent thrombectomy under local anesthesia only, without the use of sedation or general anesthesia. Consequently, the observed patterns of postoperative spontaneous BP reductions may not be fully generalizable to patients managed with conscious sedation or general anesthesia, where pharmacologic agents could independently affect hemodynamic responses. Further studies are warranted to validate our findings in broader patient populations undergoing alternative anesthesia strategies. Third, our analysis specifically focused on early spontaneous reductions in SBP following thrombectomy, rather than encompassing all directions of BP change. This focus was based on established physiological understanding: AIS typically triggers a systemic hypertensive response to support collateral perfusion, while successful reperfusion commonly leads to normalization or spontaneous reduction in BP. In contrast, persistent or increased BP after reperfusion is uncommon and may indicate atypical hemodynamic disturbances. Thus, we focused on spontaneous SBP reduction as the most clinically relevant marker of cerebral autoregulatory recovery in this context. Nonetheless, we acknowledge that excluding other BP trajectories may limit the generalizability of our findings, and future studies could explore broader BP patterns in larger, more heterogeneous populations. Finally, while this study focuses on immediate post‐thrombectomy BP dynamics, it does not address the impact of long‐term BP reductions on outcomes. To overcome these limitations, future research should include multicenter prospective trials with standardized BP monitoring protocols. Furthermore, advanced imaging modalities such as CT perfusion could provide deeper insights into the relationship between BP reductions and cerebral perfusion, aiding in the refinement of treatment strategies and optimization of patient outcomes Table 1, 2.

## 5 Conclusion

Early spontaneous SBP reduction after thrombectomy independently predicted favorable outcomes in patients with anterior LVO stroke. When combined with the early post‐thrombectomy nadir SBP, the predictive accuracy is further enhanced. These findings underscored the significance of natural BP trends and highlighted the need for personalized BP management strategies following thrombectomy.

## Author Contributions

**Jianwen Bu**: conceptualization, methodology, software, investigation, data curation, formal analysis, validation, project administration, writing ‐ original draft. **Guosen Bu**: investigation, formal analysis, supervision, project administration, writing ‐ review and editing. **Yang Zhang**: conceptualization, writing ‐ review and editing, methodology, software, formal analysis, supervision, resources, visualization, validation, funding acquisition.

## Ethics Statement

All procedures performed in studies involving human participants were in accordance with the ethical standards of the institution and with the 2008 Helsinki Declaration and its later amendments or comparable ethical standards. The study protocol followed the guidelines of the World Medical Association Declaration of Helsinki and was approved by the First Affiliated Hospital of Xinjiang Medical University's Ethics Committee (Number: LE20180223). For this retrospective study, informed consent was not required.

## Consent

We confirm that the manuscript complies with all instructions to authors. We have confirmation that authorship requirements have been met and the final manuscript was approved by all authors. This manuscript has not been published elsewhere and is not under consideration by another journal; and further, if accepted, it will not be published elsewhere.

## Conflicts of Interest

The authors declare no conflicts of interest.

## Peer Review

The peer review history for this article is available at https://publons.com/publon/10.1002/brb3.70677

## Supporting information




**Supplementary Table 1**: Comparison of Patient Characteristics Between Patients With and Without Hemorrhagic Transformation.

**Supplementary Table 2**: Univariate and multivariate regression analysis of characteristics associated with Hemorrhagic Transformation.

## Data Availability

The data that support the findings of this study are available from the corresponding author upon reasonable request.
