# Supplementary material for: Association Between Early Spontaneous Post‐Thrombectomy Blood Pressure Reduction and Clinical Outcomes in Large Vessel Occlusion Stroke
Source: Brain Behav. 2025 Jul 7;15(7):e70677. doi: 10.1002/brb3.70677 (PMC12230627; doi:10.1002/brb3.70677)
Supplement: Supplementary file 1 — Supplementary Table 1: Comparison of Patient Characteristics Between Patients With and Without Hemorrhagic Transformation. [file BRB3-15-e70677-s002.docx]

# Supplementary Table 1: Comparison of Patient Characteristics Between Patients With and Without Hemorrhagic Transformation

| **Characteristics** | **HT**  **(n=120)** | **No HT**  **(n=364)** | **P value** |
| --- | --- | --- | --- |
| Age, mean (SD), y | 73.2 ± 11.7 | 71.5 ± 12.0 | 0.417 |
| Male, n (%) | 63 (52.5) | 192 (52.7) | 0.782 |
| Hypertension, n (%) | 102 (85.0) | 304 (83.5) | 0.678 |
| No prior hypertension | 45 (37.5) | 133 (36.5) |  |
| Controlled hypertension | 58 (48.3) | 204 (56.0) |  |
| Uncontrolled hypertension | 17 (14.2) | 27 (7.4) |  |
| Diabetic mellitus, n (%) | 21 (17.5) | 56 (15.4) | 0.521 |
| Atrial fibrillation, n (%) | 20 (16.7) | 39 (10.7) | 0.148 |
| Previous stroke, n (%) | 18 (15.0) | 44 (12.1) | 0.447 |
| Chronic kidney disease, n (%) | 11 (9.2) | 31 (8.5) | 0.847 |
| New York Heart Association Class III–IV, n (%) | 5 (4.2) | 15 (4.1) | 0.779 |
| Autoimmune disease, n (%) | 3 (2.5) | 11 (3.0) | 0.912 |
| BP-altering medications, n (%) | 13 (10.8) | 42 (11.5) | 0.748 |
| Baseline NIHSS, median (IQR) | 15 (12–19) | 12 (9–16) | < 0.001 |
| Nadir SBP within 30 min post-thrombectomy, median (IQR), mmHg | 146 (138–154) | 134 (125–144) | < 0.001 |
| ΔSBP, median (IQR), mmHg | 12 (7–19) | 22 (16–28) | < 0.001 |
| Collateral score 0–1, n (%) | 72 (60.0) | 159 (43.7) | 0.004 |
| Occlusion site, n (%) |  |  | 0.941 |
| Terminal ICA | 48 (40.0) | 142 (39.0) |  |
| M1 MCA | 72 (60.0) | 222 (61.0) |  |
| ASPECTS, median (IQR) | 8 (7–9) | 9 (8–10) | 0.003 |
| Stroke etiology, n (%) |  |  | 0.788 |
| Large artery atherosclerosis | 50 (41.7) | 121 (33.2) |  |
| Cardioembolism | 52 (43.3) | 122 (33.5) |  |
| Other determined | 2 (1.7) | 7 (1.9) |  |
| Undetermined | 16 (13.3) | 114 (31.3) |  |
| rCBF < 30%, median (IQR), mL | 30 (22–38) | 21 (12–31) | 0.005 |
| Tmax > 6s volume, median (IQR), mL | 67 (48–85) | 62 (45–82) | 0.418 |
| mTICI 2b/3, n (%) | 107 (89.2) | 308 (84.6) | 0.201 |

Abbreviation: HT, hemorrhagic transformation; SD, standard deviation; NIHSS, National Institutes of Health Stroke Scale; IQR, interquartile range; SBP, systolic blood pressure; ΔSBP, change in systolic blood pressure; ICA, internal carotid artery; MCA, middle cerebral artery; ASPECTS, Alberta Stroke Program Early CT Score; rCBF, relative cerebral blood flow; Tmax, time-to-maximum; mTICI, Modified Thrombolysis in Cerebral Infarction. P-values were calculated using Pearson’s chi-square test or Fisher’s exact test as appropriate; values equal to or near 1.000 indicate results from Fisher’s exact test with no observed group differences.
