# Supplementary material for: Association Between Early Spontaneous Post‐Thrombectomy Blood Pressure Reduction and Clinical Outcomes in Large Vessel Occlusion Stroke
Source: Brain Behav. 2025 Jul 7;15(7):e70677. doi: 10.1002/brb3.70677 (PMC12230627; doi:10.1002/brb3.70677)
Supplement: Supplementary file 2 — Supplementary Table 2: Univariate and multivariate regression analysis of characteristics associated with Hemorrhagic Transformation. [file BRB3-15-e70677-s001.docx]

# Supplementary Table 2. Univariate and multivariate regression analysis of characteristics associated with Hemorrhagic Transformation

|  | **n** | **Unadjusted** | | **Adjusted** | |
| --- | --- | --- | --- | --- | --- |
|  |  | **OR (95% CI)** | **P** | **OR (95% CI)** | **P** |
| Baseline NIHSS (per point increase) | 484 | 1.12 (1.06-1.18) | < 0.001 | 1.10 (1.03-1.17) | 0.004 |
| Nadir SBP within half an hour post-procedure (per 10 mmHg increase) | 484 | 1.45 (1.23-1.71) | < 0.001 | 1.32 (1.12-1.62) | 0.001 |
| ΔSBP (per 10 mmHg decrease) | 484 | 0.68 (0.56-0.82) | < 0.001 | 0.72 (0.59-0.89) | 0.002 |
| Collateral score |  |  |  |  |  |
| 0-1 | 231 | Ref |  | Ref |  |
| 2-3 | 253 | 0.48 (0.31-0.73) | 0.001 | 0.52 (0.33-0.83) | 0.006 |
| ASPECTS (per point increase) | 484 | 0.81 (0.70-0.94)) | 0.005 | 0.85 (0.72-0.99) | 0.041 |
| rCBF <30% (per 10 mL increase) | 484 | 1.25 (1.08-1.44) | 0.003 | 1.18 (1.01-1.38) | 0.042 |

Abbreviations: OR, odds ratio; CI, confidence interval; NIHSS, National Institutes of Health Stroke Scale; ΔSBP, change in systolic blood pressure; ASPECTS, Alberta Stroke Program Early CT Score; rCBF, relative cerebral blood flow.
